# Efficacy of next-generation endoscopy training simulator in improving international trainee endoscopic submucosal dissection skills

**DOI:** 10.1055/a-2840-7240

**Published:** 2026-04-23

**Authors:** Takeshi Uozumi, Seiichiro Abe, Reona Kawamura, Yasuhiko Mizuguchi, Ryoji Ichijima, Satoru Nonaka, Haruhisa Suzuki, Yutaka Saito

**Affiliations:** 1 Endoscopy Division 70317 National Cancer Center HospitalChuo-kuTokyoJapan; 2 Division of Science and Technology for Endoscopy 70317 National Cancer Center HospitalChuo-kuTokyoJapan; 3 Division of Gastroenterology and HepatologyDepartment of MedicineNihon University School of MedicineTokyoJapan

**Keywords:** Quality and logistical aspects, Training, Endoscopy Upper GI Tract, Endoscopic resection (ESD, EMRc, ...)

## Abstract

**Background and study aims:**

Off-the-job training (OFF-JT) ensures that all trainees receive a standardized level of ESD training, regardless of the clinical workload or the regional context. This study aimed to evaluate the efficacy of OFF-JT using a next-generation laptop-based mock-up endoscopy simulator (NLMES) in improving endoscopic submucosal dissection (ESD) skills among international trainees.

**Methods:**

This study was a single-center, prospective pilot study employing a crossover design. International trainees in Group A underwent designated NLMES training and ESD observation during the early phase (Days 1–14), followed by ESD observation during the late phase (Days 15–28), whereas those in Group B followed the reverse schedule. On Days 1, 15, and 29, the trainees performed ESD using a biomaterial-free ESD model, and the ESD skills were assessed at each time point. The primary endpoint was the between-group comparison of improvement in dissection speed on Day 15.

**Results:**

A total of 10 international trainees were enrolled in this study. On Day 15, the improvement in dissection speed was greater in Group A than in Group B, although the statistical difference was not significant (6.40 mm ^2^ /min; 95% confidence interval [CI] 0.50–12.31] vs. -0.76 mm ^2^ /min; 95% CI -8.91 to 7.39], *P* = 0.087). The within-group comparison of dissection speed between before and after NLMES training demonstrated significant differences in both groups.

**Conclusions:**

OFF-JT using NLMES might be a promising approach improving international trainee ESD skills. Dissection speed increased during the period when NLMES training was received, highlighting the importance of OFF-JT.

## Introduction

Endoscopic submucosal dissection (ESD) was developed in Japan several decades ago 1 . ESD is now widely utilized as a minimally invasive treatment for gastrointestinal neoplasms not only in Japan but also in Western countries 2 3 . Although ESD enables en-bloc resection of large superficial lesions, it is a technically demanding procedure that requires extensive training for skill acquisition 4 .

There are two approaches for acquiring and improving ESD skills: on-the-job training (OJT), which consists of endoscopic procedures in clinical settings, and off-the-job training (OFF-JT), which consists of practice in non-clinical settings, such as workshops or simulators 5 6 7 . OJT is an effective approach for learning real-world application of ESD techniques, particularly in settings where trainees receive appropriate supervision and guidance 7 . In Japan, ESD traditionally has been taught using a master-apprentice model, which begins with learning in gastric neoplasms before proceeding to more technically challenging lesions 6 . Although this OJT approach has been successful in East Asian countries, it has not been applicable in Western countries due to limited opportunities to perform gastric ESD under expert supervision 8 .

**Fig. 1:**
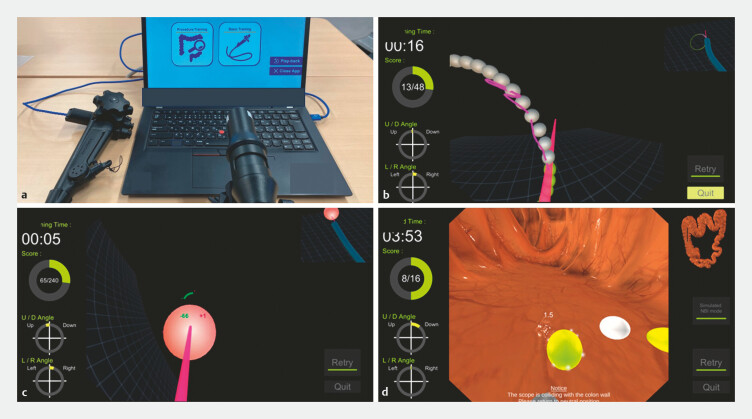
Next-generation laptop-based mock-up endoscopy simulator. **a** The NLMES is shown. **b** Trace the line. A total of six sessions were performed every day. **c** Fix the center. A total of three sessions were performed every day. **d** ESD marking. A total of three sessions were performed every day.

To address this challenge in ESD skill acquisition, OFF-JT is gaining attention 9 . OFF-JT ensures that all trainees receive a standardized level of ESD training, regardless of clinical workload or regional context. The National Cancer Center Exploratory Oncology Research and Clinical Trial Center (NCC-EPOC) has developed a next-generation laptop-based mock-up endoscopy simulator (NLMES; ENDONIX, Olympus, Tokyo, Japan) through an industry-academia collaboration with Olympus ( Fig. 1 a). The NLMES allows trainees to practice fundamental endoscopy skills, including torque and tip angulation, as well as coordination of these movements. The simulator provides equal learning opportunities regardless of trainee background and serves as a valuable tool for trainees to acquire essential skills to perform ESD. However, objective evidence supporting its educational efficacy―particularly for international trainees―remains unclear. This study aimed to evaluate efficacy of OFF-JT using NLMES in improving ESD skills among international trainees.

## Methods

### Study design

This single-center, prospective pilot study with crossover design was conducted at the National Cancer Center Hospital (NCCH) between January 2024 and January 2025. Inclusion criteria were as follows: 1) international trainees who participated in the International Endoscopy Instructional Program at the NCCH; 2) experience with at least 100 esophagogastroduodenoscopies; 3) experience with fewer than three ESD cases; and 4) observation of more than 10 expert ESD procedures at the NCCH. Trainees who stayed at the NCCH for less than 1 month were excluded because more than 1 month was necessary to complete the designated training.

Trainees were alternately assigned to the early NLMES training group (Group A) or the late NLMES training group (Group B) in a 1:1 ratio. Those assigned to Group A underwent the designated NLMES training and ESD observation during the early phase (Days 1–14), followed by ESD observation alone during the late phase (Days 15–28). Trainees assigned to Group B underwent ESD observation alone during the early phase (Days 1–14), followed by ESD observation NLMES training and ESD observation during the late phase (Days 15–28). On Days 1, 15, and 29, the trainees performed ESD using a biomaterial-free ESD model (G-Master, KOTOBUKI MEDICAL, Tokyo, Japan) 9 , and their ESD skills were assessed at each time point ( **Supplemental Fig. 1** ). Data on Day 1 were used as their baseline ESD skills.

According to the Ethical Guidelines for Medical and Biological Research Involving Human Subjects, institutional review board (IRB) approval is not required for studies aimed at evaluating acquisition or improvement of technical skills using a biomaterial-free training model that does not involve animal or human tissues. The research ethics consultation service at the NCCH confirmed that IRB approval was not necessary for the present study, which did not involve clinical data or patient information. Before enrollment, all trainees were provided an explanation of the study objectives and the outline of the study. Oral informed consent was obtained, and all trainees were given the opportunity to withdraw their consent at any time.

### International endoscopy instructional program and NLMES training

Demonstration of next-generation laptop-based mock-up endoscopy simulator training and video of representative trainees whose endoscopic submucosal dissection skills were improved after training.Video 1

International trainees participating in the International Endoscopy Instructional Program can observe screening, surveillance, and endoscopic treatment on weekdays. Lectures by Japanese expert endoscopists are occasionally offered, providing international trainees with opportunities to learn advanced endoscopy skills. International trainees who do not hold a medical license to practice in Japan are not allowed to perform any medical procedures. Duration of the International Endoscopy Instructional Program varies from 2 weeks to 1 year, depending on individual needs and backgrounds.

In the present study, trainees performed two basic training tasks and one procedure training task using the NLMES each day for 2 weeks (Days 1–14 in Group A and Days 15–28 in Group B). Designated tasks required approximately 30 minutes per day to complete. During the remaining part of the day, the trainees participated in the International Endoscopy Instructional Program. Details of the NLMES training are described below and a demonstration of the NLMES training is available in the Video 1 .

“Trace the line” is a basic training task to learn how to control up/down and right/left tip angulation by pointing a laser at a row of virtual targets ( Fig. 1 b).

“Fix the center” is a basic training task to learn how to coordinate torque and tip angulation by keeping a target at the center of the view field ( Fig. 1 c).

“ESD marking” is a procedure training task to exercise endoscopic maneuvering which simulates ESD marking ( Fig. 1 d).

During each basic training session, the total movement distance of torque and tip angulation, along with task completion times, were recorded in the NLMES system. Data from NLMES training were used to investigate the correlation between improvements in NLMES training outcomes and ESD skills.

### ESD Using a biomaterial-free model

To evaluate ESD skills, trainees performed ESD using a biomaterial-free ESD model on Days 1, 15, and 29. During ESD, the trainees used a single-channel upper gastrointestinal endoscope (GIF-H290T, Olympus, Tokyo, Japan), an electrosurgical unit (VIO-3, Erbe Elektromedizin GmbH, Waldhoernlestrasse, Germany), and an electrosurgical knife (Dual knife-J, KD-655U, Olympus, Tokyo, Japan). The target lesion was 20 mm in size and located in the lesser curvature of the middle gastric body of the biomaterial-free ESD model, which has been reported to provide high similarity to real clinical conditions, regarding anatomical orientation and the feeling of endoscope manipulation 9 . In this study, the basic strategy of ESD was as follows. After marking, a partial circumferential mucosal incision was initially made on the near side of the lesion to expose the submucosal layer. Submucosal dissection was then initiated under adequate submucosal lifting. After sufficient dissection, the circumferential incision was completed and the remaining submucosal tissue was dissected to achieve en-bloc resection. To minimize the influence of the tutor on outcomes, all procedures were supervised by a single Japanese expert endoscopist (TU), Board Certified Fellow of the Japan Gastroenterological Endoscopy Society. Trainees were allowed to receive advice from the expert endoscopist when they encountered difficulties during ESD. However, no hands-on assistance was permitted and the procedures had to be completed by the trainee alone. ESD was terminated if the procedure could not be completed within 100 minutes.

### Outcome measurements and definitions

Dissection speed was measured as a quality metric for trainee ESD skills. Dissection speed was defined as the area of the resected specimen divided by procedure time (mm ^2^ /min). Area of the resected specimen was calculated using the following formula: (major axis [mm] / 2) × (minor axis [mm] / 2) × 3.14. Procedure time was defined as the time interval from initiation of local injection to completion of specimen removal. Rates of ESD completion, en-bloc resection, and perforation were also measured to evaluate ESD skills. Improvement in dissection speed on Day 15 or Day 29 was defined as the difference in dissection speed for each trainee between Day 1 and Day 15 or between Day 1 and Day 29, respectively. The primary endpoint was the between-group comparison of improvement in dissection speed on Day 15. The secondary endpoint was the within-group comparison of dissection speed. In addition, the between-group comparison of improvement in dissection speed on Day 29 was investigated. Exploratory analyses were also conducted to identify factors associated with improved dissection speed.

### Statistical analysis

This was a pilot study; therefore, a formal sample size calculation could not be performed to determine sufficient power. The predefined sample size was set at 10 participants, based on historical data indicating that approximately 10 international trainees who participated in the International Endoscopy Instructional Program stayed in Japan for more than 1 month annually.

Welch’s t-test was used to compare independent continuous variables, whereas the paired t-test was used to compare related continuous variables. We also calculated 95% confidence intervals (CIs) for dissection speed at each time point. Scatter plots were used to visualize the association between improvement in NLMES training outcomes and dissection speed, which was evaluated by Spearman’s rank correlation coefficient. All *P* values were two-sided and statistical significance was set at *P* < 0.05. All statistical analyses were performed using R version 4.3.3 (R Foundation for Statistical Computing, Vienna, Austria).

## Results

### Characteristics of trainees from international countries

A total of 10 international trainees, six male and four female, were enrolled. Median age was 34 years (range, 28–36) in Group A and 28 years (range, 28–29) in Group B. Nationalities were Spanish (n = 3), Portuguese (n = 1), and Chinese (n = 1) in Group A and Spanish (n = 4) and Ukrainian (n = 1) in Group B. All trainees in Group A specialized in gastroenterology, whereas Group B comprised four gastroenterologists and one oncologist. In the overall cohort, one trainee had experience, with one ESD procedure performed, whereas the remaining trainees had no ESD experience. Baseline endoscopic experience was not significantly different between the two groups ( Table 1 ).

**Table:** **Table 1** Characteristics of international trainees (n = 10).

	Total	Group A	Group B
Age, median (range), years	29 (28–35)	34 (28–36)	29 (28–29)
Sex, male/female, n	6/4	3/2	3/2
Nationality, n			
Spanish	7	3	4
Ukrainian	1	0	1
Portuguese	1	1	0
Chinese	1	1	0
Specialty, n			
Gastroenterology	9	5	4
Oncology	1	0	1
EGD, n			
1–500	4	2	2
501–1000	4	2	2
≥1001	2	1	1
CS, n			
1–500	5	2	3
501–1000	2	1	1
≥1001	3	2	1
EMR, n			
1–10	6	2	4
11–50	0	0	0
≥51	4	3	1
ESD, n			
0	9	4	5
1	1	1	0
CS, colonoscopy; EGD, esophagogastroduodenoscopy; EMR, endoscopic mucosal resection; ESD; endoscopic submucosal dissection.			

### ESD outcomes

All ESD procedures on Days 1, 15, and 29 were completed without hands-on assistance. The ESD completion and en-bloc resection rates were 100% and there were no perforations in either group.

### Between-group comparison of improvement in dissection speed

Baseline mean dissection speed on Day 1 was not significantly different between Groups A and B (12.24 mm ^2^ /min [95% CI 4.50–19.98] vs. 13.39 mm ^2^ /min [95% CI 7.29–19.49], *P* = 0.754) on Day 1 ( Table 2 ).

**Table:** **Table 2** Dissection speed on Days 1, 15, and 29.

		Group A	Group B	*P* value
Day 1	Mean, mm ^2^ /min [95% CI]	12.24 [4.50–19.98]	13.39 [7.29–19.49]	0.754
Day 15	Mean, mm ^2^ /min [95% CI]	18.64 [10.96–26.33]	12.63 [8.74–16.53]	0.10
Day 29	Mean, mm ^2^ /min [95% CI]	27.39 [19.69–35.09]	18.36 [13.93–22.79]	< 0.05
CI, confidence interval.				

Mean dissection speeds on Day 15 were 18.64 mm ^2^ /min in Group A and 12.63 mm ^2^ /min in Group B, respectively ( Table 2 ). On Day 15, the improvement in dissection speed in Group A was greater than in Group B, although the statistical difference was not significant (6.40 mm ^2^ /min [95% CI 0.50–12.31] vs. -0.76 mm ^2^ /min [95% CI -8.91–7.39], *P* = 0.087).

Mean dissection speeds on Day 29 were 27.39 mm ^2^ /min in Group A and 18.36 mm ^2^ /min in Group B, respectively ( Table 2 ). On Day 29, improvement in dissection speed in Group A was significantly greater than in Group B (15.15 mm ^2^ /min [95% CI 9.45–20.85] vs. 4.97 mm ^2^ /min [95% CI -0.93–10.87], *P* = 0.009).

### Within-group comparison of dissection speed

In Group A, comparison of dissection speed between Day 1 and Day 15 was significant (12.24 mm ^2^ /min [95% CI 4.50–19.98] vs. 18.64 mm ^2^ /min [95% CI 10.96–26.33], *P* = 0.039). Comparison of dissection speed between Day 15 and Day 29 was also significant (18.64 mm ^2^ /min [95% CI 10.96–26.33] vs. 27.39 mm ^2^ /min [95% CI 19.69–35.09], *P* = 0.004).

**Fig. 2:**
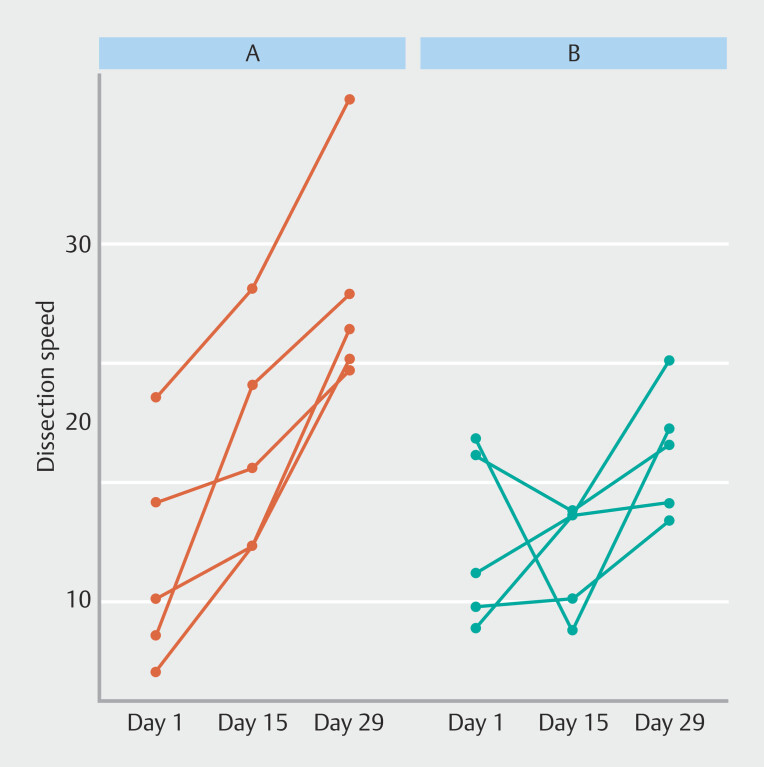
Line graph showing dissection speed of all trainees. **a** Group A. **b** Group B.

In Group B, there was no significant differences in dissection speed between Day 1 and Day 15 (13.39 mm ^2^ /min [95% CI 7.29–19.49] vs. 12.63 mm ^2^ /min [95% CI 8.74–16.53], *P* = 0.808). However, comparison of dissection speed between Day 15 and Day 29 was significant (12.63 mm ^2^ /min [95% CI 8.74–16.53] vs. 18.36 mm ^2^ /min [95% CI 13.93–22.79], *P* = 0.038) ( Fig. 2 and Video 1 ).

### Correlations between improvement in NLMES training outcome and dissection speed

**Fig. 3:**
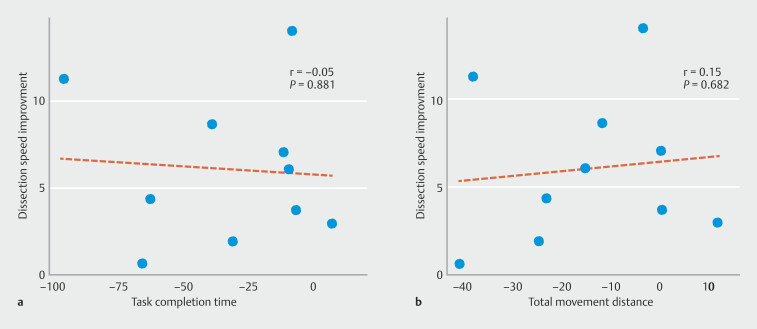
Scatter plot between the NLMES training outcome and dissection speed. Improvement in dissection speed was determined using the data obtained before and after training (Days 1–15 in Group A and Days 15–29 in Group B). **a** Correlation between the task completion time and dissection speed for each trainee. **b** Correlation between the task completion time and dissection speed for each trainee.

Dissection speed was not significantly correlated with completion time or total movement distance during “fix the center” (r = -0.05; *P* = 0.88 and r = 0.15, *P* = 0.682, respectively) ( Fig. 3 ). Moreover, dissection speed was not significantly correlated with task completion time or total movement distance during “trace the line” (r = 0.25, *P* = 0.517 and r = 0.15, *P* = 0.682, respectively).

**Fig. 4:**
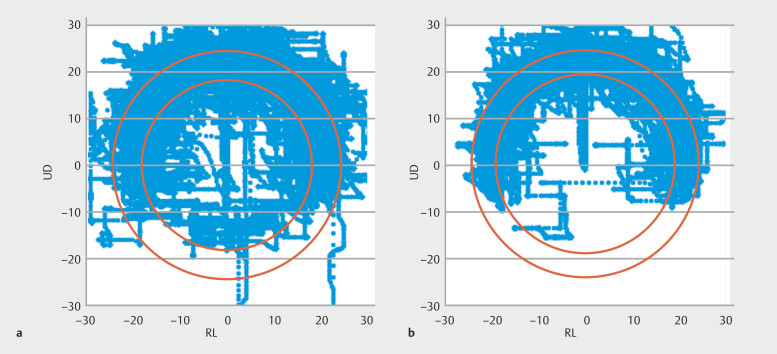
Visual summary of total movement distance. **a** First day. **b** Last day.

A visual summary of the change in total movement distance from the first day to the last day of NLMES training is shown in Fig. 4 .

## Discussion

This is the first study evaluating efficacy of OFF-JT using NLMES in improving ESD skills among international trainees. Improvement in dissection speed on Day 15 was greater in Group A than in Group B and the within-group comparison of dissection speed between before and after NLMES training revealed significant differences in both groups. These results suggest that OFF-JT using NLMES contributed to improved ESD skills, although a statistically significant difference was not observed for the primary endpoint.

This study had two advantages in evaluating efficacy of the OFF-JT using NLMES. First, dissection speed was assessed using a biomaterial-free ESD model. Dissection speed is widely utilized to measure the ESD learning curve and recognized as a quality metric of ESD skills 10 11 12 13 . However, dissection speed evaluated in clinical settings is affected by factors that are difficult to measure, such as organ deformation and respiratory motion. To address this limitation, we utilized a biomaterial-free ESD model that allowed consistent and reproducible replication of lesion size and location, thereby enabling more objective assessment of ESD skills. Second, trainees in this study did not perform any clinical duties during the study period other than the OFF-JT. Clinical duties, including screening, surveillance, or even ESD, can be a confounding factor in skill acquisition 5 14 ; however, in this study, these factors were eliminated because the trainees did not hold Japanese medical licenses. These settings allowed more accurate evaluation of OFF-JT using NLMES, with analyses revealing the importance of OFF-JT.

In the present study, we employed a crossover design, with both groups completing the same training program by Day 29. Considering this study design, we initially hypothesized that improvement in dissection speed on Day 29 would be comparable between the two groups. Surprisingly, however, improvement in dissection speed was significantly greater in Group A than in Group B, which might be attributed to a carryover effect due to absence of a washout period. In endoscopy training, observing expert procedures is widely recognized as an effective educational approach 11 . However, our findings suggest that efficacy of observational learning may be influenced by trainee prior hands-on experience. Specifically, observing expert procedures after appropriate training may enhance the educational impact. These findings suggest that timing of observational learning during endoscopy training program should be reconsidered.

Previous studies have reported that a higher number of therapeutic endoscopy procedures is associated with improvements in dissection speed 12 . However, opportunities for performing ESD are limited for international endoscopists, posing a challenge in gaining sufficient experience 15 . Therefore, identifying alternative factors that might contribute to improving ESD skills is meaningful. Although the present study failed to demonstrate correlations between the NLMES outcomes and ESD skills, they do not negate the importance of OFF-JT. There is no doubt about the importance of proficient coordination of torque and tip angulation for performing ESD. Given that NLMES facilitates development of fundamental endoscopic skills, it might be a valuable training tool for international trainees who have limited opportunities for hands-on ESD experience, particularly in the early phase of their training.

This study has several limitations. First, NLMES-based OFF-JT primarily targets technical aspects of endoscopic manipulation. It does not fully capture other essential components of real-world ESD performance, including lesion assessment, procedure planning, and intraoperative judgment. Because this study did not involve any clinical data, further study is warranted to verify that the improvements observed in this training setting translate into improved clinical outcomes. Second, this study was a pilot study, which was not based on a formal sample size calculation. Given that our findings suggesting the potential utility of OFF-JT using NLMES, robust evaluation in future studies with an appropriate sample size is warranted. Third, the ESD model was designed using a lesion located on the lesser curvature of the middle gastric body, which may be technically demanding for novice endoscopists and could have affected the appropriateness of the model for assessing ESD skills.

## Conclusions

In conclusion, OFF-JT using NLMES might be a promising approach for improving international trainee ESD skills. Dissection speed increased during the period when NLMES training was received, underscoring the importance of OFF-JT. The significantly better improvement of dissection speed on Day 29 observed in the group receiving early NLMES training suggests that early hands-on experience may enhance efficacy of observational learning.

## Supplementary Material

Supplementary Material Zusatzmaterial

## References

[1] Ono H Kondo H Gotoda T Endoscopic mucosal resection for treatment of early gastric cancer Gut 2001 48 225 229 10.1136/gut.48.2.225 11156645 PMC1728193

[2] Draganov PV Aihara H Karasik MS Endoscopic submucosal dissection in North America: A large prospective multicenter study Gastroenterology 2021 160 2317 INF 33610532 10.1053/j.gastro.2021.02.036 PMC8783061

[3] Farhat S Chaussade S Ponchon T Endoscopic submucosal dissection in a European setting. A multi-institutional report of a technique in development Endoscopy 2011 43 664 670 10.1055/s-0030-1256413 21623560

[4] Saito Y Abe S Inoue H How to perform a high-quality endoscopic submucosal dissection Gastroenterology 2021 161 405 410 10.1053/j.gastro.2021.05.051 34089735

[5] Toba T Ishii T Sato N Effectiveness of a novel ex vivo training model for gastric endoscopic submucosal dissection training: a prospective observational study conducted at a single center in Japan Clin Endosc 2025 58 94 101 10.5946/ce.2024.108 39489604 PMC11837555

[6] Oda I Odagaki T Suzuki H Learning curve for endoscopic submucosal dissection of early gastric cancer based on trainee experience Dig Endosc 2012 24 129 132 10.1111/j.1443-1661.2012.01265.x 22533768

[7] Ohata K Ito T Chiba H Effective training system in colorectal endoscopic submucosal dissection Dig Endosc 2012 24 84 89 10.1111/j.1443-1661.2012.01272.x 22533759

[8] Oyama T Yahagi N Ponchon T How to establish endoscopic submucosal dissection in Western countries World J Gastroenterol 2015 21 11209 11220 10.3748/wjg.v21.i40.11209 26523097 PMC4616199

[9] Mitsui T Yoda Y Sunakawa H Development of new gastric endoscopic submucosal dissection training model: A reproducibility evaluation study Endosc Int Open 2022 10 E1261 e1267 10.1055/a-1845-5556 36118647 PMC9473824

[10] Yoshida M Kakushima N Mori K Learning curve and clinical outcome of gastric endoscopic submucosal dissection performed by trainee operators Surg Endosc 2017 31 3614 3622 10.1007/s00464-016-5393-9 28039646

[11] Draganov PV Chang M Coman RM Role of observation of live cases done by Japanese experts in the acquisition of ESD skills by a western endoscopist World J Gastroenterol 2014 20 4675 4680 24782619 10.3748/wjg.v20.i16.4675 PMC4000503

[12] Zhang X Ly EK Nithyanand S Learning curve for endoscopic submucosal dissection with an untutored, prevalence-based approach in the United States Clin Gastroenterol Hepatol 2020 18 580 INF 31220645 10.1016/j.cgh.2019.06.008

[13] Khalaf MA Ayoub F Staggers KA Learning curve for endoscopic submucosal dissection (ESD) in the United States: Large, untutored, single-operator experience Endosc Int Open 2024 12 E905 e913 10.1055/a-2337-3865 39055261 PMC11272410

[14] Mitsui T Sunakawa H Yoda Y Novel gastric endoscopic submucosal dissection training model enhances the endoscopic submucosal dissection skills of trainees: a multicenter comparative study Surg Endosc 2024 38 3088 3095 10.1007/s00464-024-10838-3 38619558

[15] Ge PS Thompson CC Aihara H Development and clinical outcomes of an endoscopic submucosal dissection fellowship program: early united states experience Surg Endosc 2020 34 829 838 10.1007/s00464-019-06836-5 31111209

