# Supplementary figures and images for: Efficacy of next-generation endoscopy training simulator in improving international trainee endoscopic submucosal dissection skills
Source: Endosc Int Open. 2026 Apr 23;14:a28407240. doi: 10.1055/a-2840-7240 (PMC13305365; doi:10.1055/a-2840-7240)

Supplemental Figure 1 Schema of the study flow.

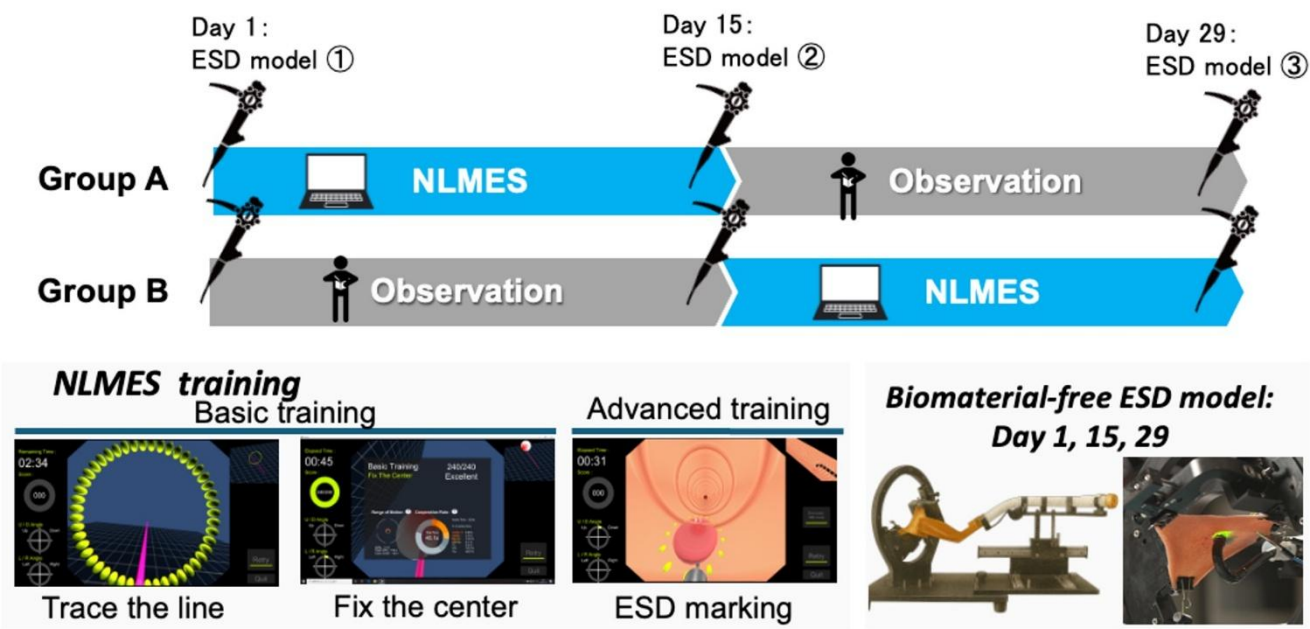

Supplement: Supplementary file 2 — Supplementary Material [file 10-1055-a-2840-7240_28445339.pdf]
